# Relationship between hyperglycemia, antioxidant capacity and some enzymatic and non-enzymatic antioxidants in African patients with type 2 diabetes

**DOI:** 10.1186/s13104-017-2463-6

**Published:** 2017-03-29

**Authors:** Constant Anatole Pieme, Jérôme Antony Tatangmo, Gustave Simo, Prosper Cabral Biapa Nya, Vicky Jocelyne Ama Moor, Bruno Moukette Moukette, Francine Tankeu Nzufo, Borgia Legrand Njinkio Nono, Eugene Sobngwi

**Affiliations:** 1 Department of Biochemistry and Physiological Sciences; Faculty of Medicine and Biomedical Sciences, University of Yaounde I, P.O. Box 1364, Yaounde, Cameroon; 2 Department of Biochemistry, Faculty of Science, University of Dschang, P.O. Box 67, Dschang, Cameroon; 3 Department of Biochemistry, Faculty of Science, University of Yaounde I, P.O. Box 812, Yaounde, Cameroon; 4 Department of Internal Medicine, Faculty of Medicine and Biomedical Sciences, University of Yaoundé I, P.O. Box 1364, Yaounde, Cameroon

**Keywords:** Oxidative stress, Total Antioxidant Capacity, Type 2 diabetes, Malondialdehyde

## Abstract

**Background and purpose:**

Studies demonstrate that free radicals are involved in the pathogenesis of diabetic complications. The aim of this study was to determine the implication of total antioxidant capacity (TAC) and some enzymatic and non-enzymatic antioxidants as suitable biomarkers of diabetic complications risk factors.

**Methods:**

A total of 90 patients (70 patients with or without diabetic complications +20 normal healthy) were examined by evaluating the level of lipid peroxidation, nitrogen monoxide (NO), fasting blood glucose, glycated haemoglobin (HbA1c), enzymatic and non-enzymatic antioxidants using standard spectrophotometric methods.

**Results:**

The fasting blood glucose and HbA1c levels were respectively 2.05 and 2.32 times higher in the group of patients with diabetes and complications (DPWC) compared to those of healthy persons. A statistically higher level of malondialdehyde (MDA), NO and TAC was observed in a group of patients with diabetes and complications compared to those without complications (DPNC). A significant positive correlation was found between catalase (CAT) and fasting blood glucose while a significant and negative correlation was noted between reduced glutathione (GSH) and fasting blood glucose. Also was noted a significant relationship between HbA1c and other markers of oxidative stress.

**Conclusions:**

The results suggest that the plasma levels of CAT, TAC and reduced glutathione could give information on the risk of developing complications of diabetes, considering that the modification of these biomarkers levels were associated with oxidative stress.

## Background

Diabetes in all their forms are characterised by either a hyperglycemia or a relative or absolute lack of insulin action. Type 2 diabetes mellitus (DM) has similar physio-pathological characteristics with type 1 DM but differs in etiology [1]. As an endocrinological disease diabetes is associated with several metabolic alterations including hyperglycemia and high level of oxidative stress. The later plays an important role in the development of diabetes complications both in the microvascular and cardiovascular systems [1]. Oxygen free radical generated from glycosylation, auto-oxidation of glycation products, changes of the tissue content and/or the activity of the antioxidant defence systems are some mechanisms involved oxidative stress in diabetics patients [2]. Free radicals not trapped by anti-oxidants during the period of oxidative stress are known to disturb endothelial dependent vasorelaxation, stimulate growth factors, induce the expression of adhesion molecules, promote blood coagulation and contribute to the formation of advanced glycosylation end products [3]. Several by-products of lipid peroxidation found in a higher level in the serum of patients with diabetes, have been correlated with the development of complications of diabetes [4, 6]. Reactive oxygen species (ROS) have been involved in the induction oxidative damage of several macromolecules including lipids, nucleic acids and proteins in patients with diabetes [7]. Most of these side effects can be prevented by antioxidants [8, 9]. Several **s**tudies have provided evidences of oxidative damage of different macromolecules and their impact on antioxidant/pro-oxidant systems in type 2 diabetic patients. A number of antioxidants exists in the cells either enzymatic (superoxide dismutase, glutathione peroxidase and catalase) or non-enzymatic (as glutathione and uric) as scavengers of ROS, to prevent oxidative damage of biological membranes [7, 10, 11]. Beside these antioxidants found in the cells, natural antioxidants exist from vegetables and most of them including vitamin A, vitamin C, vitamin E and carotenoids. To improve our knowledge on the pathogenesis of some metabolic diseases, several clinical and experimental studies have been undertaken in order to understand the role of the oxidative stress in the pathogenesis of diabetes [3, 5]. These investigations have described that free radicals generated during this oxidative process is involved in genesis of diabetes, plays significant role in its development of diabetes and complications [1, 4, 7, 12–15]. In such a context, it appears important to evaluate the level of oxidative stress parameters in blood as biomarkers that can be used to identify and prevent the risk of promoting diabetic complications. Up till now, the methods used to detect the status of oxidative stress in patients at clinical level are scarce in literature. Although some useful methods are available to investigate the oxidative profile, they are unfortunately not applicable for clinical diagnosis. However, some of these methods can be used to better understand the appearance of complications that occur during the development of some metabolic diseases such as diabetes. In this light, it seems important to associate the blood levels of TAC, GSH and fasting blood glucose in order to understand what happens in patients with type 2 diabetes with complications compared to healthy persons. The measure of antioxidant capacity considers the cumulative action of all the antioxidants present in plasma and body fluids, thus providing an integrated parameter rather than the simple sum of the measurable antioxidants. The capacity of the known and unknown antioxidants and their synergistic interaction is therefore assessed, thus giving an insight into the delicate balance in vivo between oxidants and antioxidants. In this study serum levels of enzymatic and non-enzymatic antioxidant markers were determined in three groups of persons (healthy persons, diabetic patients without complications (DPNC) and diabetic patients with complications (DPWC) and discussed the relationships between these markers.

### Population of study

A total of 70 patients with type 2 diabetes (40 diabetic patients without complications (DPNC) and 30 diabetic patients with complications (DPWC) and 20 healthy persons (as controls) were recruited. These diabetic patients with/without complications were regularly followed up at the Diabetology and Obesity Unit of the Central Hospital of Yaoundé. Participants who accepted to take part in the study were recruited on a consecutive basis and enrolled after completing and signing the standardised data collection forms which included socio-demographic characteristics and medical history. We included in the study, patients who had the following criteria for DPNC: age ranging between 30 and 90 years old with glycated haemoglobin (HbA1c) >6.2% of total Hb; fasting blood glucose comprised between 0.8 and 1.2 g/l; post prandial glycemia lower than 2 g/l. The abstaining participants who were diagnosed or whose medical history revealed the presence of glaucoma, cataracts, retinopathy; neuropathy, nephropathy, hypertension, hyperlipidemia and ketoses were classified among DPWC. The two groups of diabetic patients were treated with insulin (hormonotherapy) or drugs glucose-lowering effects such as sulfonylureas, biguanides.

These patients were matched age and sex wise with healthy persons without diabetes. All diabetic patients and healthy people affected with other disease or under medication which can affect oxidative stress markers were excluded in our study. Lifestyle, habits and previous medical records were collected from each subject. The control group of subjects was made of healthy volunteers recruited either at the University or in different families. A written informed consent, approved by the National Ethical Committee was obtained for all subjects (Reference: CBI/231/ERCC/CAMBIN-Protocole N^o^ 024, 29th/11/2013).

### Availability of data and materials

We obtained the consent to publish the data from the participants reporting individual patient’s data.

## Methods

### Samples collection

From each fasted individual who accepted to participate to the study and signed the consent form, venous blood samples were collected into two different tubes, a sodium fluoride plain and an EDTA coated tubes. Samples contained in plain tubes were stored for 30 min at 4 °C prior to centrifugation at 3000 rpm for 5 min to obtain the serum samples which were stored at −20 °C. These serum samples were subsequently used to determine antioxidant and pro-oxidant parameters. The samples contained in EDTA coated tubes were used to determine the glycated hemoglobin (HbA1c ≥ 6.2 0%) and plasma glucose concentration.

### Determination of pro-oxidant markers

The fasting blood glucose and glycated hemoglobin were determined with commercial kits (Human, Germany). Malondialdehyde (MDA), an end product of unsaturated fatty acid peroxidation can react with thiobarbituric acid (TBA) to form a colored complex called thiobarbituric acid-reactive substances (TBARS) [16]. Results were expressed in µmol per mg of protein.

The nitrogen monoxide (NO) concentration in the serum was measured using the Griess’ reaction, by adding 100 µl of Griess’ reagents (0.1% naphthylethylendiamide dihydrochloride in H_2_O and 1% sulfanilamide in 5% concentrated H_3_PO_4_; vol. 1:1) to 100 µl of samples. The optical density at 550 nm was then obtained in a spectrophotometer. The nitrite levels were calculated by using sodium nitrite as a standard and expressed as in µmol [17].

### Measurement of antioxidant markers

The total antioxidant capacity (TAC) was determined by the Ferric Reducing Ability of Plasma (FRAP) method in which a colourless ferric tripyridyltriazine complex is reduced to a blue ferrous complex by the antioxidants in the serum. Briefly, a mixed solution of 50 µl of serum and 50 µl of distilled water was added to 900 µl of FRAP reagent and incubated at 37 °C for 25 min. The change in absorbance at 593 nm is directly related to the total reducing power of electron donating antioxidants present in the serum [18]. The results were expressed in µmol per mg of protein.

The reduced glutathione level was determined by the Ellman’s method [19]. This method was based on the development of yellow colour when 5,5′-dithio-bis-2-nitrobenzoic (DTNB) is added to compound containing sulphydryl groups. The colour developed was read at 412 nm in the spectrophotometer. Results were expressed as µmol/mg protein.

The superoxide dismutase (SOD) activity of the serum was determined according to the method described by Misra and Fridovich [20]. The method is based on the inhibition of epinephrine auto-oxidation. The absorbance was read at 480 nm against a blank and the SOD activity was expressed as U/mg of protein.

The activity of serum CAT was determined according to the method described by Sinha [21]. The results were expressed as U/mg of protein.

### Statistical analysis

The results obtained were presented as mean ± SEM. The analysis of data was conducted using Kruskal–wallis test and Dunnett’s multiple test (SPSS program version 18.0). The software Graph Pad Instat 3 was used to achieve the correlation between the groups. The differences were considered as significant at *P* < *0.05*.

## Results

**Fig. 1:**
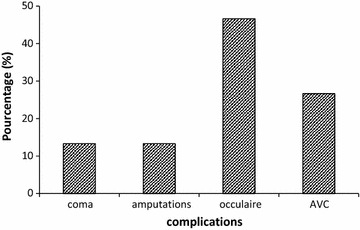
Different complications found on DPVC

The characteristics of all the population involved in this study are shown in Table 1. Among all the participants in the different groups, men were more represented than women with a sex ratio of 1.09. However this value varied from 1.10 to 1.5 depending on the group of patients. The age of the patients was between 30 and 90 years with an average of 54 ± 3 years. The most represented age group was between [40 and 60] for healthy people (75%) and DPNC (60%) and between [50 and 70] for DPWC (73%). Among the risk factors studied, alcohol consumption was the most represented either in healthy people or patients with diabetes with/without complications. The complications mostly found in the DPWC were ocular disorders (46.6%) and stroke (26.6%) (Fig. 1).

**Table 1:** Characteristics of the sample population

	Control N = 20 (%)	DPNC N = 40 (%)	DPWC N = 30 (%)
Gender			
Male	60	52.5	53.3
Female	40	47.5	46.66
Age			
30< age <40	5	10	3.33
40< age <50	40	27.5	16.66
50< age <60	35	32.5	36.66
60< age <70	10	20	36.66
>70	10	10	6.66
Smoking	0	5	23.33
Alcohol	60	32.5	76.66
Family history	50	25	53.33
Hypertension	5	20	36.66

*DPNP* diabetic patients without complications, *DPWC* diabetic patients with complications

### Variation of clinical parameters

Glycated hemoglobin and fasting blood glucose levels determined to assess the glycemic status of patients are represented in Table 2. These results show a significant increase in glycosylated hemoglobin and blood glucose in diabetic patients with/without complications compared to healthy persons. HbA1c and fasting blood glucose levels of diabetic patients with complications are respectively 2.05 and 2.32 times higher compared to those of healthy persons. No significant change in BMI in patients of different groups while lipid peroxidation index increased from healthy patients to DPWC.

**Table 2:** Variation of clinical parameters

	Control (mean ± SD) N = 20	DPNC (mean ± SD) N = 40	DPWC (mean ± SD) N = 30	*P* value
HbA_1_C (%)	4.75 ± 0.11^a^	8.20 ± 0.44^b^	9.73 ± 0.52^c^	P < 0.05
Fasting blood glucose (g/l)	1.15 ± 0.05^a^	2.11 ± 0.13^b^	2.67 ± 0.17^c^	P < 0.05
BMI (kg m^2^)	29.03 ± 5.23^a^	27.20 ± 5.28^a^	25.92 ± 5.78^a^	P > 0.05

Results are expressed as mean ± SD, Values subscripted with different letters are significantly different in the same line

*DPNC* diabetic patients without complications, *DPWC* diabetic patients with complications, *BMI* body mass index, In the same column, values subscripted with different letter are significantly different

### Variation of stress oxidative markers

The results of this study showed the changes in both oxidant and antioxidant statuses of patients. As shown in Table 3, the mean value of SOD activity was higher and significant in the control group compared to the group of patients with diabetes. This value decreased significantly from DPNC to DPWC with the respective values of 0.38 to 0.24 U/mg of protein. Similar results were noted with CAT activity. However, the CAT activity of the control group was significantly lower compared to that of patients with diabetes (Table 3). The results of the total antioxidant capacity decreased significantly (*P* < 0.05) from the healthy persons (210.48 µmol/mg of protein) to DPWC (171.35 µmol/mg of protein). However, no significant difference was observed between DPNC and DPWC groups. MDA is a low molecular weight aldehyde that can be produced from free radical attack on polyunsaturated fatty acids of biological membranes and the determination of this marker is used for monitoring lipid peroxidation in biological samples. Serum MDA and nitrite levels as well as lipid peroxidation index (LPI) were significantly higher in diabetic patients with and without complications indicating the free radical mediated oxidative damage of lipids. The concentration of TAC and NO were significantly higher in a group of DPWC who smoke and drink alcohol compared to DPNC (Table 4). A significant and negative relationship was noted between fasting glucose and glutathione in DPNC while a significant and positive correlation was observed between fasting glucose and CAT, FRAP and glutathione respectively in DPWC and healthy people (Table 5). Moreover, a significant and positive relationship was also noted between HbA1c, NO, MDA and GSH while the negative and significant correlation was noted between HbA1c and TAC demonstrating that oxidative stress may affect HbA1c.

**Table 3:** Variation of oxidant and antioxidant parameters

	Control (mean ± SD) N = 20	DPNC (mean ± SD) N = 40	DPWC (mean ± SD) N = 30
MDA (µmol/mg of protein)	0.28 ± 0.03	0.29 ± 0.02	0.32 ± 0.03
NO_2_^−^ (µmol)	191.41 ± 11.54	216.07 ± 8.96	227.38 ± 10.15
TAC (µmol/mg of protein)	210.48 ± 6.22	160.46 ± 7.65^*^	171.35 ± 6.43^*^
CAT (U/mg of protein)	28.34 ± 2.67	37.37 ± 1.78^*^	35.19 ± 2.37^*^
SOD (U/mg of protein)	0.41 ± 0.04	0.38 ± 0.03^*^	0.24 ± 0.01^*^
GSH (µM/mg of protein)	6.77 ± 0.59	9.78 ± 0.58^*^	9.79 ± 0.73^*^
LPI (MDA/TAC)	0.180	0.181	0.186

Results are expressed as mean ± SD

*DPNP* diabetic patients without complications, *DPWC* diabetic patients with complications, *LPI* lipid peroxidation index, *MDA* malondialdehyde, *CAT* catalase, *SOD*: superoxide dismutase, *GSH* reduced glutathione, *NO*_*2*_ nitrite oxide

* P < 0.05 significant compare with the control

**Table 4:** Comparison of oxidant and antioxidant parameters of alcohol and smoking patients

Parameters	DPNC (mean ± SD)	DPWC (mean ± SD)	*P values*
Alcohol			
SOD (U/mg of protein)	0.4190 ± 0.2484	0.2347 ± 0.07693	0.0314
TAC (µmol/mg of protein)	141.93 ± 57.322	184.00 ± 42.294	0.0327
Smoking			
NO_2_ (µM)	199.60 ± 67.633	244.86 ± 16.249	0.0345

*DPNP* diabetic patients without complications, *DPWC* diabetic patients with complications, *SOD* superoxide dismutase, *TAC* total antioxidant capacity

**Table 5:** Correlation between antioxidant and clinical parameters in different groups of patients

	Control N = 20		DPNC N = 40		DPWC N = 30	
	*r* ^*2*^	*P value*	*r* ^*2*^	*P value*	*r* ^*2*^	*P value*
Fasting blood glucose and CAT	0.252	0.285	−0.151	0.340	0.460	0.011
Fasting blood glucose and glutathione	0.134	0.573	−0.540	0.001	0.025	0.897
TAC and glutathione	0.563	0.010	0.122	0.440	−0.059	0.758
HbA1 and NO	0.392	0.04	0.76	0.003	0.123	0.004

*DPNP* diabetic patients without complications, *DPWC* diabetic patients with complications, *CAT* catalase, *TAC* total antioxidant capacity, *NO* Nitrite oxide

P < 0.05 is significant

## Discussion

Diabetes does not only alter the metabolism of carbohydrates, lipids and protein, but also the chemistry of these macromolecules. Poorly controlled diabetes accelerates the chemical modification of proteins and their functions which could lead to the development of diabetic complications. In this context, several hypotheses have been emitted in order to understand the origin of the complications observed in diabetic patients. These hypotheses include mitochondria damage, mitochondrial defect in oxidative phosphorylation, increased formation of advanced glycation end products (AGES), increased activity of the polyol pathway, hypoxia, alteration of lipoprotein metabolism, increased protein kinase C activity, alteration of growth factors and cytokine, activities and increased oxidative and reductive stress [15, 22]. Although oxidative stress appears as one of the metabolic events associated to diabetes and its complications the precise mechanisms by which oxidative stress may accelerate the development these complications are still not well understood [7]. Oxidative stress seems to be increased in a system where the rate of free radicals production is increased and/or the antioxidant mechanisms are impaired [22].

The present study examined the variation in both extra and intracellular antioxidants, oxidant status, fating blood glucose as well as HbA1c in a group of diabetic patients without or with complications. The glycated hemoglobin (HbA1c fraction) which estimates the fasting blood glucose over a period of three months is useful and considered as a reference parameter in monitoring glycemic control in diabetic patients. Our results showed the increase in fasting blood glucose and HbA1c levels in a group of diabetic patients with complications (DPWC) compared to those without complications and healthy persons. Indeed hyperglycemia causes tissue damage through 5 major mechanisms: (1) increased flux of glucose and other sugars through the polyol pathway; (2) increased intracellular formation of AGEs (advanced glycation end products); (3) increased expression of the receptor for AGEs and its activating ligands; (4) activation of protein kinase C (PKC) isoforms and (5) the over activity of the hexosamine pathway [13]. Previous studies have shown that an increase in glucose levels induces diabetes, the overproduction of oxygen free radicals which consequently increases the protein and lipid oxidation. Malondiadehyde (MDA) is highly toxic by-products partly produced by oxidation and derived from lipid. MDA reacts both irreversibly and reversibly with proteins and phospholipids with profound effects. In this study, high statistical significant differences were observed in the levels of MDA in these cases. MDA was higher in the case of DPNC and DPWC groups than in the control group. Similar results were found in previous studies [23, 24]. The observed high levels of plasma MDA in diabetic patients reflect lipid peroxidation which is the consequence of oxidative stress. The increase in the level of MDA correlates with hyperglycemia in these patients because of self-oxidation of glucose and could generate free radicals. A significant correlation exists between the biomarkers of oxidative stress and fasting blood glucose in DPWC and DPNC. Our study demonstrated a significant positive correlation between fasting blood glucose and CAT activity while significant negative association was demonstrated between fasting blood glucose and glutathione. Nitrogen monoxide (NO) is an important vascular target for ROS. Ion Superoxide, NO and the peroxynitrite formed are sources of hydroxyl radicals that could cause endothelial damage [2]. Increased levels of NO observed in DPNC and DPWC compared to healthy patients could be explained by the fact that hyperglycemia in diabetic patients affects the vascular endothelium and the associated vascular tone by affecting NO levels. In the diabetic state, the endothelium is exposed to high concentrations of glucose and this likely leads to some of the vascular abnormalities which result due to glucose toxicity. The endothelial dysfunction associated with diabetes has been attributed to lack of bioavailable NO due to reduced ability to synthesise NO from l-arginine [25]. NO is a candidate for mediating hyper filtration and the increased vascular permeability induced by diabetes. The higher level of NO in these groups correlates with the reduction of total antioxidant capacity. The reduction of the total antioxidant capacity in the presence of higher levels of lipid peroxidation in serum of patients with type 2 diabetes indicates the existence of oxidative stress. The significant correlation between HbA1c and serum with NO content found in the present study confirms that hyperglycemia control may directly influence NO synthesis. It is well known that the expression of NO action largely depends on its relative level and on its interaction with superoxide anions. These results support the findings that vascular dysfunction due to exposure to pathologically high d-glucose concentrations may be caused by impairment of the NO pathway and increased oxidative stress accompanied by altered glucose metabolism.

The synergistic effect of antioxidants in human plasma is known to provide greater protection against free radical aggression than any single antioxidant alone. In addition to the total antioxidant capacity (TAC), the enzyme antioxidant activities to trap the free radicals generated under normal or pathological conditions were evaluated by measuring SOD, CAT and reduced glutathione level. The high concentration of superoxide dismutase causes impairment of endothelial isoform of nitrogen monoxide synthase (eNOS) by triggering advanced glycation end products and poly (ADP-ribose) polymerase [26].

Reduced glutathione (GSH), a non-enzymatic antioxidant plays an excellent role by protecting cells from oxidative damage keeping up the cellular levels of the active forms of vitamins C and E by neutralizing the free radicals. The increase in the GSH level in DPNC and DPWC may be related to increase activity of glutathione peroxidase which acts by neutralizing free radicals produced during the complication of the disease. This extracellular non-enzymatic antioxidant delays or inhibits the oxidative process through different mechanisms. Antioxidant enzyme levels are particularly sensitive to oxidative stress and both increase or decrease these have been reported in different disease states in which an enhancement of oxygen species is a cause or a consequence of the disease [7]. The significant variation in CAT and SOD activities in the serum of diabetic patients with and without complications was also noted. The increase in CAT activity may be a respond to an induction of the enzyme caused by the high level of its substrate (organoperoxides and H_2_O_2_) [24]. This observation may results in a number of deleterious effects due to the accumulation of superoxide radicals and hydrogen peroxides [27]. Our results confirm that diabetes, alcohol and smoking are associated with an increase of oxidative stress, which results in higher serum concentration MDA and nitrogen monoxide radical or in lower level of SOD activity in DPWC group. To the best of our knowledge very few studies demonstrated these findings. However, it has been reported that excessive use of alcohol can contribute to increase BMI [27, 28]. Our results of SOD and CAT indicate a relationship between these two enzymes which may play an important role in the effective control of oxidative stress without the variation of one or the other being sufficient independently [7]. The ratio CAT/SOD is considered to be a biomarker of the glycemic control while the MDA/FRAP ratio is a biomarker of lipid peroxidation [7, 28]. These two ratios are considered as the risk factors for the development of diabetes complications. Increase in lipid peroxidation products (as indicated by the level of MDA) due to the decrease activity of most of the antioxidant enzymes is in line with previous reports [24, 29]. Hence complications of diabetes may be the result of this high level of free radicals (increase in the level of MDA and peroxidation index) and the reduction in antioxidant defences (SOD and total antioxidant capacity). This study provides evidence of oxidative damage of different molecules and its impact on antioxidant/pro-oxidant system in type 2 diabetic patients with or without complications.

## Conclusion

Our studies showed that the levels of fasting blood glucose and HbA1c, MD and NO were higher in a group of diabetic patients compare the control group. Except for SOD, HbA1c and fasting blood glucose, there was no significant difference between the two groups of diabetic patients. The increase of fasting blood glucose affected the levels of reduced glutathione and catalase in the group of diabetic patient. The correlation between different markers can provide additional information on the risk of diabetic complications.

## Abbreviations

BMI: body mass index
CAT: catalase
DM: diabetes mellitus
DPNC: group of diabetic patients without complications
DTNB: 5,5′- dithio-bis-2-nitrobenzoic
DPWC: group of diabetic patients with complications
EDTA: ethylene diamine tretra acetic acid
GSH: reduced glutathione
MDA: malondialdehyde
NO: nitrgen monoxide
H_3_PO_4_: phosphoric Acid
eNOS: nitric oxide synthase
ROS: reactive oxygen species
SOD: superoxide dismutase
TAC: total antioxidant capacity
TBA: thiobarbituric acid
TBARS: thiobarbituric acid-reactive substances
